# A novel loss-of-function mutation in NRAP is associated with left ventricular non-compaction cardiomyopathy

**DOI:** 10.3389/fcvm.2023.1097957

**Published:** 2023-02-06

**Authors:** Zhongman Zhang, Kangkang Xu, Lianfu Ji, Han Zhang, Jie Yin, Ming Zhou, Chunli Wang, Shiwei Yang

**Affiliations:** ^1^Department of Cardiology, Children’s Hospital of Nanjing Medical University, Nanjing, China; ^2^Nanjing Key Laboratory of Pediatrics, Children’s Hospital of Nanjing Medical University, Nanjing, China

**Keywords:** cardiomyopathy, left ventricular non-compaction, *NRAP*, zebrafish, RNA-seq

## Abstract

**Background:**

The nebulin-related-anchoring protein (*NRAP*) gene encodes actin-associated ankyrin. Few studies reported the association of the *NRAP* gene with cardiomyopathy. Thus, the genetic role of this gene in cardiomyopathy remains to be investigated.

**Methods:**

The clinical data of the rare case of left ventricular non-compaction (LVNC) were collected and analyzed. Whole-exome sequencing (WES) was performed on related family members. Western blot was used to detect the effect of mutation on the *NRAP* protein expression. The effect of the c.259delC variant on myocardial development was further evaluated in a zebrafish model.

**Results:**

A novel homozygous frameshift mutation c.259delC of *NRAP* was found in the proband with LVNC. It was found that c.259delC decreased the expression of *NRAP* by Western blot. In the zebrafish model, the heart development was affected while knocking out the *NRAP* gene, which showed pericardial edema. The pathological manifestations were uneven hypertrophy, disordered arrangement of cardiomyocytes, enlarged intercellular space, and loose muscle fibers. RNA-sequencing (RNA-seq) showed that the expression of genes related to heart development decreased significantly, and the *NRAP* gene mutation could participate in biological processes (BPs) such as myocardial contraction, cell adhesion, myosin coarse filament assembly of striated muscle, myosin complex composition, and muscle α-actin binding.

**Conclusion:**

We identified a rare case of LVNC associated with a novel homozygous NRAP frameshift variant. This study further strengthened the evidence linking mutations in the NRAP gene with LVNC, providing a new clue for further study of LVNC. NRAP may be one of the pathogenic genes of cardiomyopathy.

## Introduction

Left ventricular non-compaction (LVNC) was first described in 1990 (1) and was known as a unique type of inherited cardiomyopathy characterized by excessive trabecular meshwork and deep intertrabecular recesses in the LV wall (2, 3). The prevalence of LVNC in the pediatric age is around 0.14% (4), and individual differences in clinical manifestations are relatively large from being asymptomatic to heart failure. LVNC is a polygenic heterogenic cardiomyopathy inherited as an autosomal dominant or X-linked recessive disorder, although autosomal recessive and mitochondrial (maternal) inheritance also occur (5). The etiology and specific pathogenesis of LVNC have not been fully elucidated. The molecular genetic analysis uncovered causal mutations for LVNC in many genes, including *NKX2-5*, *TAZ*, *LMNA*, *MYH7*, *ACTC1*, *LDB3*, *TNNT2*, and *MYBPC3*, which encode mitochondrial proteins, sarcomere proteins, and cytoskeleton (5, 6). However, the types of these pathogenic genes and mutations are complex, and there are interactions between various pathogenic genes, which makes the mechanism study quite complicated.

Therefore, for LVNC, the precise pathogenic mechanism from molecular component and cellular component (CC) to clinical phenotype is still unknown. At present, clinical drug treatment cannot reverse the progression of LVNC, and the treatment is mainly symptomatic. It becomes especially important to discover the causative gene of LVNC and clarify the functional mechanism to define genotype-to-phenotype associations and provide a theoretical basis for gene-targeted therapy.

Nebulin-related-anchoring protein (*NRAP*) is a multi-domain cytoskeletal protein specifically expressed at the terminal bundles of actin filaments at the myotendinous junction of skeletal muscle and the intercalated disk of cardiac muscles (7). It is located on chromosome 10q25.3 and consists of 42 exons, encoding 1,730 amino acids, and including a Lin-11, Isl-1, and Mec-3 (LIM) domain followed by 11 simple repeats and 5 super repeats each consisting of 7 repeats (8–10). The N-terminal LIM domain was confirmed to interact with α-actin and talin, which links integrin to the actin cytoskeleton at the cell surface, while the C-terminal super-repeat domain interacts with actin and vinculin. The simple repeat domain of *NRAP* interacts with α-actin, actin, the Kelch-like family member 41 (KLHL41), and muscle LIM protein (MLP) (7, 11). *NRAP* participates in the assembly process of myofibrils by interacting with the aforementioned structural proteins (12–14). At the same time, it was found that *NRAP* protein can anchor the terminal actin filaments of myofibrils to the cell membrane and transfer the tension of myofibrils to the extracellular matrix (15). In theory, the *NRAP* gene mutation may lead to musculoskeletal system disease and cardiomyopathy since *NRAP* is specifically expressed in heart and skeletal muscle. However, previous studies found that *NRAP*-related diseases are mainly concentrated in muscular diseases including myofibrillar myopathy and mitochondrial myopathy (16–18). There are few clinical reports of *NRAP* gene mutation causing cardiomyopathy.

In this study, a rare case of LVNC associated with *NRAP* gene mutation was analyzed. Through whole-exome sequencing (WES), we identified novel *NRAP* variants (c.259delC) in this family. We investigated the effects of this variant on protein expression by Western blot *in vitro*. Then, we established *NRAP*^–/–^ mutant zebrafish embryo models by clustered regularly interspaced short palindromic repeats (CRISPR)/Cas9 to study the effects of the variant on the heart development of zebrafish. Furthermore, transcriptomics analysis was performed to speculate the pathway of the *NRAP* gene regulating cardiac development, which may provide new clues for the pathogenesis of LVNC.

## Materials and methods

### Whole-exome sequencing

The study was performed according to the Ethics Committee Guidelines of the Children’s Hospital of Nanjing Medical University after communicating with the parents of the child to sign an informed consent form. Blood samples were collected *via* puncture of the cubital vein in tubes filled with ethylene diamine tetra acetic acid. Blood samples were stored at 4°C and were used to extract genomic DNA following the standard phenol–chloroform extraction method. WES was performed in the proband and his parents and young sister. More than 1.5 μg of genomic DNA from each sampled individual was sequenced using the Illumina HiSeq 2000 sequencer (Bio-Rad, Hercules, CA, USA) following the manufacturer’s procedures. Variants with allele frequencies higher than 1% in the public databases [Genome Aggregation Database (gnomAD), dbSNP, 1000 Genomes minor allele frequency (MAF) (Chinese), Exome Aggregation Consortium (ExAC), and an in-house MAF database] were filtered out. The candidate variants were validated by Sanger sequencing, and the pathogenicity of variants was annotated according to the American College of Medical Genetics and Genomics (ACMG) standards and guidelines.

### Construction of cDNAs encoding *NRAP* mutants

Wild-type (WT) full-length human *NRAP* cDNA (GenBank NM_001261463) was purchased and cloned into pcDNA3.1–3xFlag vectors using a ClonExpress Entry one-step cloning kit (Vazyme, Nanjing, China). The c.259delC *NRAP* variant was engineered into the pcDNA3.1–3xFlag-WT vector by site-directed mutagenesis using the PCR-based *Dpn*I-treatment method (Vazyme). The mutagenesis was confirmed by bidirectional sequencing.

### Cell culture and transfection

The cell line HEK-293 was maintained and transiently transfected with WT or mutant *NRAP*. The HEK-293 cells were seeded in six-well plates for transfection and maintained in culture at 37°C with 5% CO_2_ in Dulbecco’s Modified Eagle’s Medium (DMEM) supplemented with 10% fetal bovine serum (FBS). To express WT or c.259delC mutant *NRAP*, the cells were transiently transfected with plasmids of pcDNA3.1–3xFlag-WT (4 μg) or pcDNA3.1–3xFlag-c.259delC (4 μg) using Lipofectamine 2000 (8 μL) transfection reagents after the cells were 50–70% confluent. The transfected cells were incubated at 37°C for 48–72 h before performing the next experiments.

### Western blot analysis

After the indicated transfection, HEK293 cells were rapidly washed with ice-cold phosphate-buffered saline (PBS). The total proteins were extracted using RIPA Lysis Solution (Beyotime, China) and the protein concentration was quantified by the BCA kit (Beyotime, China). A total of 30 μg of total protein was fractionated in 8% polyacrylamide gel and then transferred to nitrocellulose membranes. Membranes were then blocked with tris buffered saline (TBS) with Tween-20 (TBST) containing 5% skim milk at room temperature for 1 h to block non-specific binding sites. The primary antibody against Flag (1: 3,000, Sigma Aldrich, St. Louis, MO, USA), GAPDH (1: 3,000, ProTech, Chicago, IL, USA), or Na^+^/K^+^-ATPase (1:1,000, Abcam, Cambridge, MA, USA) was incubated overnight at 4°C and then washed with TBST. The horseradish peroxidase (HRP)-labeled secondary antibody incubation was then performed at room temperature for 1 h. Antibody binding was determined by chemiluminescence reaction. Target bands were quantified using Image J software (NIH, Bethesda, MD, USA).

### Zebrafish husbandry

Experimental and husbandry procedures using zebrafish [Tübingen (TU) strain, WR] were approved by the Model Animal Research Center, Nanjing University (Nanjing, Jiangsu, China). Water temperature was maintained at 28°C with conductivity between 400 and 600 μS and pH between 7.0 and 7.5 in a 14/10 h light/dark cycle. Water was changed every day. Natural spawning was used to collect embryos which were raised at 28.5°C in an embryo medium. All experimental procedures were performed according to the protocol. All animal experiments were performed in accordance with the approval of the National Institutes of Health Guidelines for the Care and Use of Laboratory Animals.

### Generation of *NRAP* mutant by CRISPR/CAS9 and RNA microinjection

The amino acid sequences encoded by human *NRAP* and zebrafish *nrap* (ENSDARG00000009341) were compared, and it was found that the mutation positions were all in the 87aa of amino acid on the exon 4. Based on this analysis, it is recommended to design the target sequence to be knocked out on exon 4. Genome editing in zebrafish embryos was achieved using CRISPR/Cas9 with sgRNA target sequence 5′-GAATAAGTATAAGGAAGAAG-3′ designing and selecting by the CRISPR design tool. Primers containing the T7 promoter sequences, target sequences, and SgRNA skeleton sequences were synthesized and used for amplification from a gRNA template (pYSY-sgRNA plasmid). sgRNAs were transcribed *in vitro* using T7 RNA polymerase by an RNA *in vitro* transcription kit (Ambion, Austin, TX, USA). After mixing sgRNA (final concentration of about 100 ng/μL) and Cas9 protein (final concentration of 250 ng/μL), they were microinjected into zebrafish zygotes at a volume of 1 nl per embryo. The abnormal morphology of the heart and the heart rate of embryos were observed and photographed using a stereoscopic microscope. The abnormal group of zebrafish heart tissue sections was observed.

### Transcriptome sequencing analysis

In preparation for RNA-sequencing (RNA-seq), the heart tissues of adult zebrafish (>3 months old) were sorted into two groups based on their genotypes (mutant type and WT). RNA was extracted and sequenced using the Illumina NextSeq platform by Yaoshunyu Biotechnology Co., Ltd. (Nanjing, Jiangsu) to get raw data. After cleaning and trimming of low-quality reads and adaptor removal, clean reads were matched to the reference sequence and were used for further transcriptome. These high-quality reads were then mapped to the zebrafish reference genome (*Danio rerio*, GRCz11) using HISAT2. Unique mapped reads were used to quantify gene expression in each sample by reads per kilobase of transcript per million mapped reads (RPKM) arithmetic. The differentially expressed genes (DEGs) between the two groups were identified using the DESeq2 package. We carried out functional annotation analysis to recognize the DEG-enriched biochemical pathways using the Kyoto Encyclopedia of Genes and Genomes (KEGG) database^1^ and biological processes (BPs) in Gene Ontology (GO). The enrichment analyses were considered significantly enriched if Benjamini-adjusted *p*-value was less than 0.05.

### Statistical analysis

All data were presented as mean standard error. Statistical analysis was performed using analysis of variance (ANOVA) analysis followed by a Bonferroni posttest. A *P*-value of < 0.05 was considered statistically significant.

## Results

### Clinical features and genetic analysis

**FIGURE 1:**
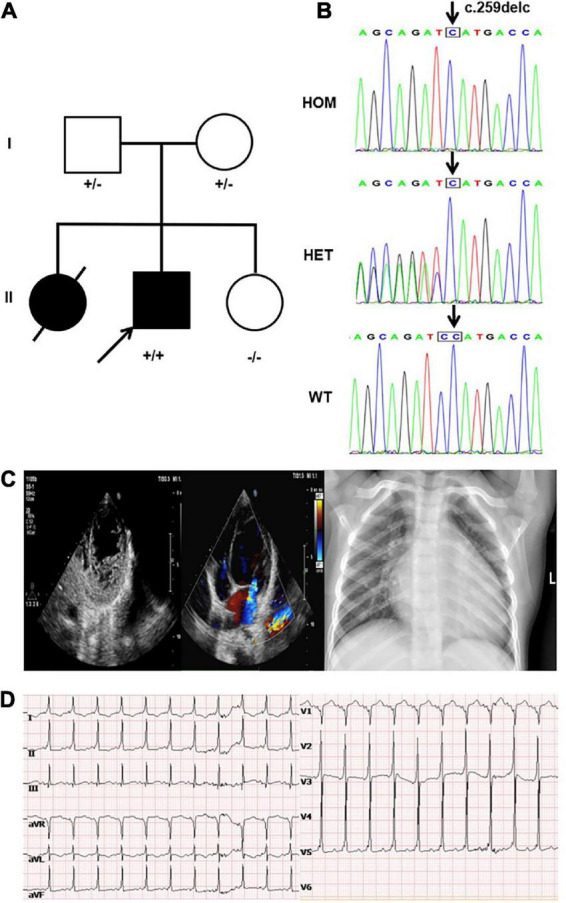
Clinical and genetic findings. **(A)** Pedigree: the proband was indicated by an arrow. The presence or absence of the nebulin-related-anchoring protein (NRAP) variant is indicated by a ± symbol. **(B)** Identification of a homozygous mutation NRAP frameshift mutation c.259delC in the proband inherited from his father and mother. **(C)** Echocardiogram showed an enlarged left atrium and ventricle of the heart, prominent trabecular meshwork, and deep intertrabecular recesses. Chest X-ray of the proband showed an enlarged left ventricular (LV) cavity. **(D)** The ECG revealed sinus tachycardia, ventricular pre-excitation, and T-wave changes in some leads.

The proband was a boy who was born *via* cesarean section with a birth weight of 4.0 kg. His parents are healthy and his mother has had three children (Figure 1A). The first child was a girl diagnosed with LVNC due to shortness of breath at the age of 2 years. She had mild weakness in muscles and died of heart failure at the age of 5 years, while no other detailed medical records or genetic data were available for her. The third child was a healthy girl. At the age of 3 years, the proband was referred to the hospital because of fatigue and shortness of breath. Physical examination revealed shortness of breath, orthopnea, systolic murmur in cardiac apex, and a slightly low first heart sound. Meanwhile, it was found that the myodynamia of his lower limbs were slightly lower. The proband had motor delays, including an unstable head and inability to roll over spontaneously, but his intellectual development was fair. His echocardiogram showed an enlarged left atrium and ventricle, prominent trabecular meshwork and deep intertrabecular recesses, mild mitral valve regurgitation, tricuspid regurgitation, pulmonary hypertension (36 mmHg), and reduced cardiac function [LV ejection fraction (LVEF): 35.5%], and the ratio of the thickness of the non-compacted layer to that of the compacted layer was 3.136 at the end of diastole. At the same time, the echocardiography was performed on the proband’s parents and his sister and no obvious abnormality was found. Chest X-ray showed an enlarged heart shadow (Figure 1C). The ECG revealed sinus tachycardia, ventricular pre-excitation, and T-wave changes in some leads (Figure 1D). He was diagnosed with LVNC and administered digoxin, furosemide, spironolactone, L-carnitine, captopril, and aspirin. Echocardiography suggested that the child’s cardiac function was slightly improved with an LVEF of 38%. After the patient was discharged from the hospital, he was readmitted to the hospital two times due to infection; his condition worsened during the outpatient clinic follow-up and died of heart failure at about 4 years old.

A novel homozygous *NRAP* frameshift mutation c.259delC (p.H87Mfs*17) was detected in the proband by using WES, and Sanger sequencing was used to validate it. The mutation caused the deletion of base C, present between bases 258 and 260 on the 4th exon, generating a histidine (H) at the 87th position of the *NRAP* gene and shifting the reading frame of the following amino acids. After 17 amino acids following the 87th amino acid, subsequently, a stop codon appeared and led to the premature termination of protein synthesis. Genetic screening showed that the mutation was inherited from his parents; in other words, proband’s parents both carried the *NRAP* heterozygous mutation (Figure 1B). This mutation had not been registered in the single nucleotide polymorphism (SNP) database of the National Center for Biotechnology Information.^2^ According to the criteria supplied by ACMG, this mutation could be classified as likely pathogenic (PVS1 + PM2) (19). Bioinformatic analysis based on PROVEAN and MutationTaster suggested that the variant was a disease-causing mutation.

### Expression of *NRAP* in HEK293 cells

**FIGURE 2:**
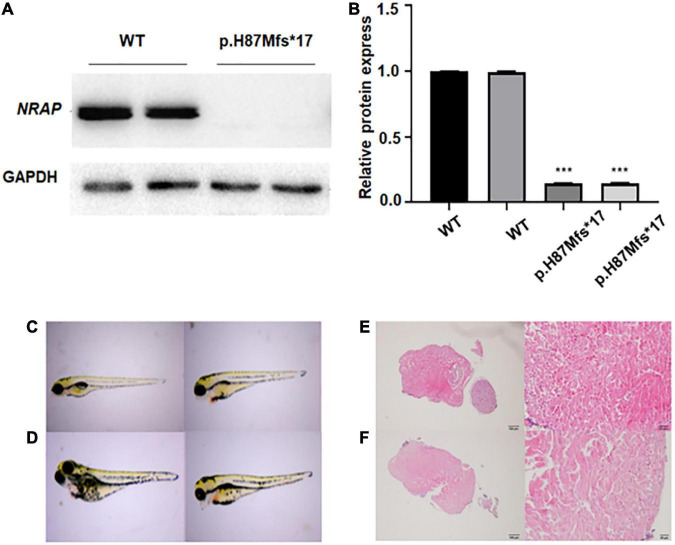
Functional study of p.H87Mfs*17 mutation in nebulin-related-anchoring protein (NRAP). **(A)** Total cell protein expressions of wild-type (WT) vs. the mutated NRAP determined by Western blotting. p.H87Mfs*17 mutation led to decreased expression of NRAP. **(B)** Quantitative analysis of total cell protein expressions of WT vs. mutated NRAP. All values are means ± SD; *n* = 6 in each group. ***P < 0.001 vs. control. **(C,D)** Images of juvenile fish with pericardial edema. Two cases were the wild-type (WT) zebrafish **(C)** and two cases carried a homozygous mutation of the NRAP allele **(D)**. **(E)** HE-stained heart tissue of normal zebrafish. **(F)** HE-stained heart tissue of zebrafish with pericardial edema.

Levels of protein expression analysis of whole-protein lysate from HEK293 cells transfected with WT or *NRAP* mutant plasmids revealed that the mutation resulted in decreased protein levels as compared with WT HEK293 cells (Figure 2A), and quantitative normalization was performed using GAPDH (Figure 2B).

### Effect of p.H87Mfs*17 mutation on pericardium development in zebrafish

To clarify whether the p.H87Mfs*17 variant affects the function of the *NRAP* and to investigate how the mutation affects heart development *in vivo*, we generated a transgenic zebrafish line expressing the corresponding *NRAP* mutation using CRISPR-Cas9 zebrafish (F1) carrying *NRAP* mutation that was self-crossbred 1 to 1 to obtain F2 embryos, and then 52 embryos were randomly selected. A total of 52 fertilized eggs were observed for pericardium development by video microscopy, and all 52 embryos developed normally at 24, 48, 72, and 96 hpf. While seven embryos with pericardial edema were observed in 52 embryos at 120 hpf. The aforementioned seven juvenile fish with pericardial edema under the microscope were collected, and the genomic DNA template was prepared for PCR amplification and sequencing. We found that two cases were the WT zebrafish (Figure 2C), two cases carried a homozygous mutation of the *NRAP* allele (Figure 2D), and three cases carried *NRAP* heterozygous alleles, suggesting that homozygous mutation may not cause death. The proportions of the three genotypes in developing malformed embryos conform to Mendel’s law of monogenic inheritance. We observed hematoxylin-eosin (HE)-stained zebrafish heart tissue with pericardial edema under the microscope, and the pathological features are uneven hypertrophy of myocardial cells, disordered arrangement, enlarged intercellular space, and loose muscle fibers (Figures 2E, F).

### Identification of DEGs and functional enrichment analysis of DEGs

To assess the DEGs, we compared gene expression differences among different sample groups. Based on the cutoff threshold (fold change > 1), a total of 838 genes were identified as DEGs, of which 471 genes were down-regulated and 342 genes were up-regulated after removing 25 DEGs belonging to unknown genes or ncRNAs. Among them, several genes related to heart development, such as *MYBPC1*, *MYBPC2A*, *TNNI2A*, *TNNT3B*, and *TBX*, were significantly down-regulated in the p.H87Mfs*17 variant group. To interpret the differentially expressed protein-coding genes, KEGG pathway and GO enrichment analyses of the variants were performed. The top 10 items of our GO enrichment analysis are shown in Supplementary Figure 1A–C. GO analysis of down-regulated DEGs revealed that the p.H87Mfs*17 variant was correlated to a significant enrichment of muscle contraction and muscle system process in BP, actin cytoskeleton, sarcomere, and myofibril in CC, and structural constituent of muscle, structural constituent of muscle, and actinin binding in molecular function (MF). The KEGG pathway enrichment suggested that the down-regulated genes particularly enriched in adrenergic signaling in cardiomyocytes, tight junction, and cardiac muscle contraction (Supplementary Figure 1D). The results demonstrated that these genes are largely involved in regulating myocardial contraction and muscle development.

## Discussion

The molecular genetic pathogenic mechanisms of LVNC are complex. Current research supported the development of LVNC which was associated with abnormal maturation and compaction in the final stages of embryonic endomyocardial morphogenesis (20). Many mutations have been identified related to hereditary LVNC, indicating the molecules and pathways causing cardiomyopathy. In this study, we identified a novel variant of the *NRAP* gene in children with LVNC.

In this study, our patient suffered from LVNC with an enlarged left atrium and ventricle and decreased heart function, and his sister died at 5 years of age due to LVNC. The *NRAP* c.259delC (p.H87Mfs*17) mutation can lead to the early appearance of the stop codon and was expected to remove about 90% of the important part of the full-length protein. The bioinformatics analysis software concluded that the mutation is a loss-of-function (LOF) mutation, which is highly pathogenic. The results of Western blotting showed that c.259delC decreased the *NRAP* protein, which suggests it could be a LOF variant too.

At the same time, combined with the literature, we speculated that the loss of protein function may further affect the systolic function of cardiomyocytes, resulting in the phenotype of LVNC. Then, we used CRISPR/Cas9 to successfully establish the *NRAP* gene knockdown zebrafish model to further explore its possible molecular mechanism. Microscopic morphological observation revealed that *NRAP*^–/–^ transgenic zebrafish affected the development of the zebrafish heart, manifesting as pericardial edema, further supporting that the mutation is a LOF variant. After that, we stained the heart tissue of zebrafish suffering from pericardial edema with hematoxylin-eosin. Uneven hypertrophy, disordered arrangement, enlarged intercellular space, and loose muscle fibers pathology were observed, which is consistent with the diagnosis of LVNC to a certain extent.

High-throughput transcriptome sequencing was performed on *NRAP*-WT and *NRAP*^–/–^ zebrafish embryos. It turned out that the expression of genes related to heart development such as *MYBPC1*, *MYBPC2A*, *TNNI2A*, *TNNT3B*, and *TBX* significantly decreased in *NRAP*^–/–^ zebrafish embryos. While the human homologous genes *MYBPC1*, *MYBPC2*, *TNNI2*, *TNNT3*, and *TBX6* are all important genes in cardiac development. The results indicated that *NRAP* gene mutation can be involved in BPs such as myocardial contraction and cell adhesion, causing cardiac disease.

The c.259delC (p.H87Mfs*17) mutation had certain effects on *NRAP*, but whether it leads to LVNC was uncertain. The main pathway leading to LVNC refers to the pathogenic changes of genes involved in the basic structural protein components of cardiac muscle, including proteins encoding sarcomeres, cytoskeletons, and ion channels. *NRAP* is a multi-domain cytoskeletal protein that is specifically expressed at the junction of cardiac intercalated disks and skeletal muscle tendons. In theory, *NRAP* gene mutation may lead to cardiomyopathy. While there are only a few reports of *NRAP* associated with cardiomyopathy, there was no report of *NRAP* associated with LVNC. In 2017, an *NRAP* gene homozygous nonsense mutation (c.4504C > T) was first discovered in a patient with dilated cardiomyopathy (DCM). The LV wall, the right ventricle, and the ventricular septum of the proband’s heart were detected by Western blot, and no *NRAP* protein was expressed in these heart tissues. It means the nonsense mutation of *NRAP* may lead to cardiac dysfunction (21). Vasilescu et al. (22) collected a countrywide cohort of 66 severe childhood cardiomyopathies from the sole center in Finland performing cardiac transplantation and identified a homozygous nonsense variant in *NRAP* (c.1344T > A, p.Y448*). The predicted protein truncation at amino acid 488 most likely leads to a complete loss of function, while they showed that the *NRAP* mRNA harboring the premature stop codon is not degraded in the patient’s heart biopsy. They believed that *NRAP* truncation can cause childhood cardiomyopathies (22). Koskenvuo et al. (23) performed a retrospective analysis of 31,639 individuals to determine the frequency of rare NRAP variants in a cohort of patients with DCM and control patients. They found that biallelic NRAP variants could explain 0.25–2.46% of all DCM cases. They believed that biallelic LOF in NRAP causes autosomal recessive DCM (23), while LVNC has substantial genetic overlap with DCM (24), suggesting that NRAP mutation may play a pathogenic role in LVNC.

Although there was evidence that *NRAP* plays an important role in myocardial architecture and sarcomere function, no variant in this gene has been conclusively linked to heart disease to date. Therefore, whether *NRAP* gene mutations directly lead to cardiomyopathy and its specific molecular mechanism need to be further studied.

In conclusion, we identified a novel variant of *NRAP*, c.259delC (p.H87Mfs*17), in patients with LVNC through WES. The results herein suggested that the variant causes a loss of function of the *NRAP* protein. Our study further strengthened the evidence linking mutations in the NRAP gene with LVNC, providing a new clue for further study of LVNC. NRAP may be one of the pathogenic genes of cardiomyopathy.

## Data availability statement

The datasets generated for this study can be found in the LOVD database with Individual ID 00430656 (https://databases.lovd.nl/shared/individuals/00430656).

## Ethics statement

The studies involving human participants were reviewed and approved by the Ethical Committee of Children’s Hospital Affiliated to Nanjing Medical University. Written informed consent to participate in this study was provided by the participants’ legal guardian/next of kin.

## Author contributions

ZZ and KX edited the manuscript. LJ contributed to the sample collection. HZ, JY, and MZ conducted and analyzed the experiments. CW and SY designed the study and performed the statistical analysis. All authors contributed to the article and approved the submitted version.

## Footnotes

1
http://www.genome.ad.jp/kegg/

2
www.ncbi.nlm.nih.gov

## Supplementary material

Supplementary Figure 1 Transcriptomic analyses. **(A–C)** Functional enrichment of the genes with Gene Ontology (GO) biological process (BPs), cellular component (CC), and molecular function (MF) enrichment. **(D)** Functional enrichment of the genes with the Kyoto Encyclopedia of Genes and Genomes (KEGG) pathway analysis.
