# Supplementary figures and images for: A novel loss-of-function mutation in NRAP is associated with left ventricular non-compaction cardiomyopathy
Source: Front Cardiovasc Med. 2023 Feb 6;10:1097957. doi: 10.3389/fcvm.2023.1097957 (PMC9940605; doi:10.3389/fcvm.2023.1097957)

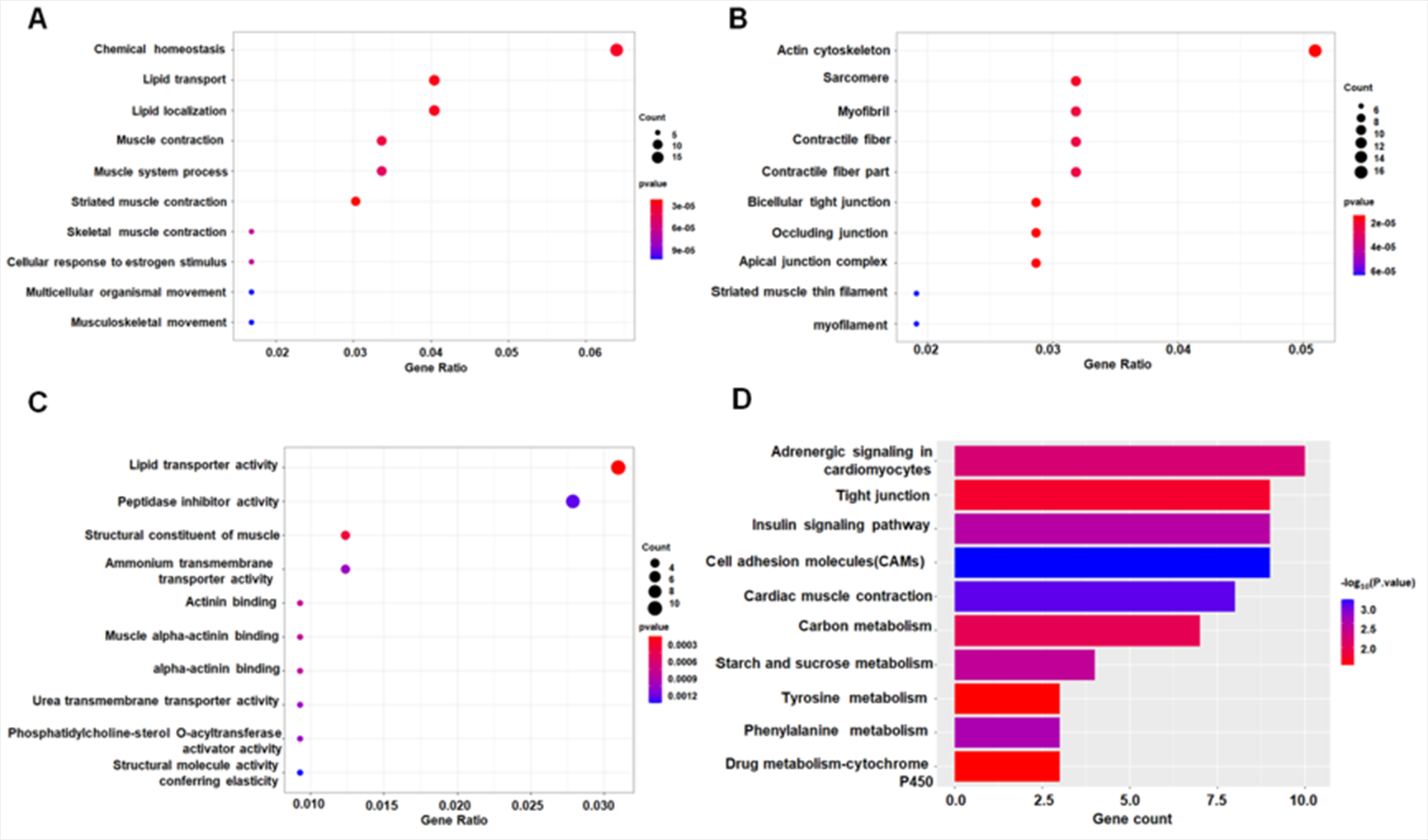

Supplement: Supplementary Figure 1 — Transcriptomic analyses. (A–C) Functional enrichment of the genes with Gene Ontology (GO) biological process (BPs), cellular component (CC), and molecular function (MF) enrichment. (D) Functional enrichment of the genes with the Kyoto Encyclopedia of Genes and Genomes (KEGG) pathway analysis. [file Image_1.TIF]
